# Immunohistochemical Coexpression of Epithelial Cell Adhesion Molecule and Alpha-Fetoprotein in Hepatocellular Carcinoma

**DOI:** 10.1155/2018/5970852

**Published:** 2018-07-19

**Authors:** Leonardo do Prado Lima, Carla Jorge Machado, João Bernardo Sancio Rocha Rodrigues, Leonardo de Souza Vasconcellos, Eduardo Paulino Junior, Paula Vieira Teixeira Vidigal, Vivian Resende

**Affiliations:** ^1^Departamento de Cirurgia, Hospital das Clínicas da Faculdade de Medicina, Universidade Federal de Minas Gerais, Belo Horizonte, MG, Brazil; ^2^Departamento de Medicina Social e Preventiva da Faculdade de Medicina, Universidade Federal de Minas Gerais, Belo Horizonte, MG, Brazil; ^3^Departamento de Patologia Clínica, Faculdade de Medicina, Universidade Federal de Minas Gerais, Belo Horizonte, MG, Brazil; ^4^Departamento de Histopatologia da Faculdade de Medicina, Universidade Federal de Minas Gerais, Belo Horizonte, MG, Brazil

## Abstract

**Background and Aim:**

The epithelial cell adhesion molecule (EpCAM) has been proposed as a marker for cancer stem cells in human hepatocellular carcinoma (HCC) as well as in the development of novel target therapies. This study aimed to investigate the immunohistochemical expression of EpCAM and alpha-fetoprotein (AFP) in HCC patients and their association with clinicopathological characteristics.

**Methods:**

This study included Child-Pugh A HCC patients undergoing curative surgical resection.

**Results:**

A significant difference was observed in the ratio between the different phenotypes (p = 0.002), identifying 12 (29.3%) EPCAM positive tumors and 29 (70.7%) negative tumors. EpCAM+ expression was associated with AFP + (OR = 12.5, 95% CI, 1.9-84.1, p<0.001). In univariate analysis, a significant association was observed between AFP+ and EPCAM+ and the serum AFP level. A diameter of ≤ 5 cm was associated with EPCAM+, while angiolymphatic invasion was associated with APF+. In a multivariate analysis, only tumors of ≤ 5 cm were significantly associated with EpCAM+ (OR = 8.7; 95%CI, 1.27-100.0; p = 0.022). The overall survival rate was 74.9%, 69.4%, 69.4%, and 53.5% at 12, 24, 36, and 48 months, respectively.

**Conclusion:**

A considerable number of patients with EpCAM+ HCC would benefit from a specific target therapy.

## 1. Introduction

Hepatocellular carcinoma (HCC) is the sixth most common cancer worldwide and the third in cancer-related mortality [1]. According to the Barcelona Clinic Liver Cancer (BCLC) criteria [2], less than 20% of all diagnosed patients are willing to undergo surgical treatment. In addition, frequent postoperative recurrence is also quite common [1, 3]. Even in the early stages, the five-year survival rate is only 55%, reaching even lower rates in later stages [4].

The so-called Cancer Stem Cells (CSC), a small distinct subpopulation of cells exhibiting consistent properties as stem cells, such as self-renewal, cell proliferation, and differentiation, have been demonstrated in HCC. These cells would be responsible for tumor initiation, as well as their biological behavioral patterns, including angiolymphatic invasion, metastasis, and recurrence [4]. Many surface proteins have been suggested as biomolecular markers of CSC in HCC, including the epithelial cell adhesion molecule (EpCAM) [4, 5]. EpCAM is a type I transmembrane glycoprotein, known as the specific cancer antigen 17-1 A, which acts as a calcium-independent homophilic cell adhesion molecule. The intracellular domain of EpCAM works directly as a transcription factor that activates c-myc, cyclin A, and cyclin E in promoting cell cycles and proliferation [6, 7].

Yamashita et al. [8] were one of the first to characterize EpCAM in the HCC cell line, demonstrating that EpCAM-expressing cells have self-renewing and differentiating properties such as stem cells. These authors proposed a new classification system using EpCAM expression and blood alpha-fetoprotein (AFP) assay to reveal different phenotypes of HCC. The importance of this classification would be to provide new perspectives on molecular pathway activation in HCC [9]. Moreover, the EpCAM antigen could be used to detect circulating tumor cells [6, 10]. It has been demonstrated that EpCAM positive cells play a relevant role in cancer progression and have been identified as a molecular biomarker for chemoresistance [7, 10–13].

The morphological characteristics, associated with the molecular study, could contribute to the identification of a specific biomarker and, potentially, the development of new molecular HCC target therapies. New drugs that act to inhibit specific biomolecular markers could become a more therapeutic option, especially in those chemoresistant tumors with high rates of relapse. In addition, most studies on the expression of EpCAM and AFP in HCC were performed in Asian populations.

In this context, the present study aimed to investigate the immunohistochemical expression of these molecular biomarkers and their association with clinicopathological characteristics in HCC patients undergoing curative surgical resection.

## 2. Method

### 2.1. Patients

This study included Child-Pugh A HCC patients undergoing curative surgical resection at the Federal University of Minas Gerais (UFMG) from 2011 to 2016. Informed consent was obtained from each recruited patient prior to surgery. This research was approved by the UFMG Research Ethics Committee (protocol number: CAAE-25010114.5.0000.5149). Patients submitted to chemoembolization and previous radiofrequency or extrahepatic metastatic disease were excluded.

### 2.2. Clinicopathological Data

The following data were prospectively collected: sex, age, comorbidities, etiology of the liver disease, preoperative serum AFP level, type of liver resection (minor, equivalent to two or fewer hepatic segments, single or multiple nodules, the largest tumor diameter (≤ 5 cm or > 5 cm), type and degree of histological differentiation (well, moderate, and poorly differentiated), angiolymphatic invasion, and TNM staging.

The diagnosis of hepatocellular carcinoma was based on clinical history, serum AFP level, imaging (ultrasound, CT scan, and/or magnetic resonance), or histopathology report. Staging followed the standards recommended by the World Health Organization (WHO) [14].

Survival data were obtained from patient medical records or by telephone contact with family members, when the doctor's appointments were interrupted without notification of death. Deaths that occurred within 30 postoperative days were excluded from the survival analysis.

### 2.3. Tumor Samples

Representative samples of 43 HCC were obtained from resected surgical specimens analyzed at the UFMG Histopathology Service. The samples were fixed in 10% formaldehyde for 24 hours, embedded in paraffin, cut into 4 *μ*m thick sections, and stained by hematoxylin and eosin (HE). Two samples were excluded from the study due to insufficient material in the paraffin block. For immunohistochemistry, the sections were deposited onto adhesive-coated glass slides.

### 2.4. Immunohistochemistry

Immunohistochemical staining for anti-EpCAM (Mouse, B302 - 323 / A3, Abcam, 1:200) and anti-alpha1 fetoprotein (Rabbit, EPR9309, Abcam, 1:50) was performed according to manufacturer instructions. Briefly, the slides were initially left in an oven at 60°C for 12 h. The sections were then deparaffinized in xylol and rehydrated in successive alcohol baths. After, epitope retrieval was performed in a citrate buffer at pH 6.0 in a vegetable steamer for 30 minutes. The endogenous peroxidase was blocked with 3% hydrogen peroxide for 15 min and proteins for 10 minutes. Next, overnight incubation with the primary antibody was conducted. After removing the antibody, the complement was placed, and the HRP conjugate (Advance HRP Polymer) was applied for 30 minutes. Staining was viewed using 3,3′-diaminobenzidine substrate-chromogen (DAB) solution followed by counterstaining with hematoxylin. For the expression of AFP, a fetal liver fragment was used as the external positive control. For the expression of EpCAM, the internal control was the positive labeling of this protein in the bile duct epithelium. PBS was substituted for the primary antibody in negative controls.

The slides were evaluated by two experienced pathologists in immunohistochemical analysis, without prior knowledge of the clinical and pathological information of the patients. The expression of AFP and EpCAM was evaluated and considered to be binary, considering a positive marking when more than 10% of the tumor cells expressed the markers in a moderate or strong form. Samples were considered negative when there was no marking or when less than 10% of the tumor expressed the proteins [5].

### 2.5. Statistical Analysis

Statistical analysis was performed using a statistical package Stata for Mac 2016. Student's* t*-test and Fisher or Chi-square tests were applied in order to verify the association of antibodies and clinicopathological variables. The magnitude of the associations was obtained by odds ratio (OR). Based on the results of the univariate analysis, variables with p values of less than 0.30 (p <0.30) were selected as candidates for multivariate model composition. In the multivariate analysis, the exact logistic regression model was used for the sequential deletion of the variables, with a p value greater than 0.10 (p>0.10). Survival curves were analyzed by the Kaplan-Meier method and comparison between groups by the Log-rank test. Deaths that occurred within 30 days of the postoperative period were excluded from the survival analysis. All statistical tests were two-sided and the level of significance was 5% (p<0.05).

## 3. Results

**Table 1** shows the demographic, clinical, and laboratory characteristics of HCC patients. This study identified 12 (29.3%) EPCAM positive tumors and 29 (70.7%) negative tumors. The EpCAM+/AFP+ phenotype was observed in 8 (66.7%) tumors, whereas EpCAM+/ AFP- was found in 4 (33.3%) tumors. The EpCAM-/AFP- phenotype was found in 25 (86.2%) tumors, while the EpCAM-/AFP+ was identified in 4 (13.8%) tumors. Serum AFP level mean was 1864.1 (SD: 9266.3); Median (IIQ), 59 (54; 66); Minimum, Maximum: 1.9, 59.900.

**Figure 1:**
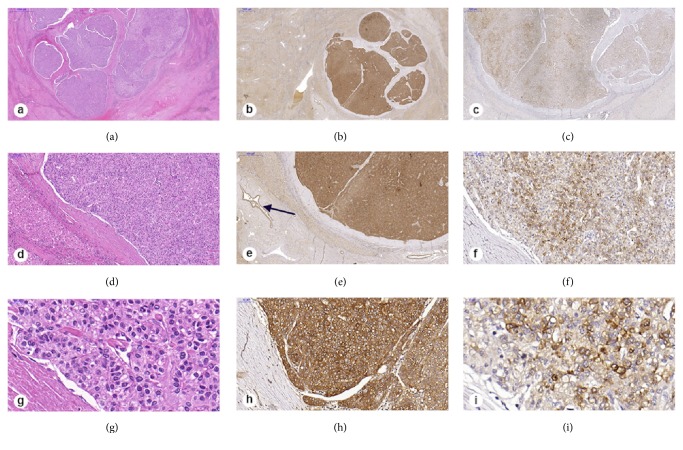
Histology of HCV-related HCC, single nodule, largest diameter =1.7 cm, well differentiated. HE (a, d, g); EpCAM+ (b, e, h) and AFP+ (c, f, i). Internal positive control for EpCAM is observed in the bile duct (arrow). Blue bars show increase in micrometers.

**Figure 1** illustrates the EpCAM+/AFP+ phenotype.

**Table 2** shows the association between the HCC immunohistochemical expression of EpCAM and AFP. A significant difference was observed in the ratio between the different phenotypes (p=0.002). The chance of occurrence of positive EpCAM expression when AFP is positive was 12.5 times the chance of positive EpCAM expression when AFP was negative (OR = 12.5, CI=95% 1.9-84.1, p <0.001).

**Table 3** shows the association between clinicopathological variables and EpCAM and AFP positivity. In a univariate analysis, a significant association was observed between the serum AFP level with EPCAM and the AFP immunohistochemical positivity. Angiolymphatic invasion was associated with APF+, while the HCC diameter ≤ 5 cm was associated with EPCAM+. In a multivariate analysis, only the tumors ≤ 5 cm were significantly associated with EpCAM+ (OR = 8.7; 95% CI, 1.27-100.0; p = 0.022). The chance of positive EpCAM expression in moderately and poorly differentiated tumors was 5.74 times greater than the chance of positive EpCAM expression in well-differentiated tumors (OR = 5.74, 95% CI, 0.93-50.3; p = 0.063). The chance of angiolymphatic invasion when alpha-fetoprotein expression was positive was 4.3 times greater than the chance when AFP was negative in HCC (OR = 4.27; 95% CI, 0.87-24.8; p = 0.079).

Five deaths (12.2%) occurred in the first 30 postoperative days. During the follow-up time (range, 12 to 48 months), 13 patients died (36%). The mean survival was 24.19 months (+/- 18.9 months), while the median was 18 months (IIQ: 9.5; 38), with a minimum of 2 months and a maximum of 48 months. None of the clinicopathological variables were associated with survival. The overall survival rate was 74.9%, 69.4%, 69.4%, and 53.5% at 12, 24, 36, and 48 months, respectively.

## 4. Discussion

HCC is the most common primary tumor of the liver, and its incidence has increased in Western countries [1, 6, 15]. In the present study, hepatitis C and B, as well as, alcoholic disease, accounted for 82.92% of the tumors, which accounts for the most frequent causes of chronic liver disease in Western countries. In addition, the predominance of men and the mean age of patients were similar to the other centers reported in the literature.

A high serum AFP level is used as diagnostic criteria for hepatocellular carcinoma (in the absence of a testicular tumor). However, about 20% to 80% of HCC patients do not have high AFP levels [16–20]. In agreement with the literature, this study observed a median of 59 ng / mL serum AFP level, with an AFP below 100 ng / mL in 63.41% of the patients. To the best of our knowledge, the present study is unique, as it uses the immunohistochemical expression of AFP in the tumor, as well as the blood level of this marker.

EpCAM expression is described only in bile duct epithelium but not in the mature hepatocyte membrane [21]. EpCAM positive expression has been found in 15.9% to 48.7% of all hepatocellular carcinomas [9, 22–25]. The EpCAM protein was positive in 50.9% of the confluent multinodular type HCCs, in 23.9% of the single nodules, and in 28.4% of the single nodules with extracapsular growth [26]. If we consider only the single nodules in this previous report, the result obtained in our study (29.3% of EpCAM+) is in accordance with these authors. Similar to Guo et al. [5], in this study, EpCAM expression was significantly more frequent in patients with elevated serum AFP levels (p = 0.006).

EpCAM expression was more frequent in HBV-related HCC than in those with other etiologies [6]. Although this relationship does not have a well-defined mechanism, one hypothesis is that HBV promotes hepatocarcinogenesis through the development of cancer stem cells through the activation of Wnt/*β*-catenin signaling pathways, thus leading to an overexpression of EpCAM [10]. In the present study, the expression of EpCAM and AFP was not related to the viral etiology.

Bae et al. (2012) [27] detected a positive expression of EpCAM in 41% of the total HCC cases. Among the 35 small size cases (2 cm), EpCAM expression was detected in 19 (54%) tumors. It is believed that this molecule plays an important role in the early stages of tumor development due to its stem cells properties. In this study, tumors ≤ 5 cm were those which expressed more EpCAM, with significant results in univariate and multivariate analyses. It can therefore be hypothesized that as the tumor grows and differentiates, the cells lose the phenotype of stem cells and fail to express EpCAM. However, complementary studies need to be performed to prove this hypothesis.

Angiolymphatic invasion was associated with AFP expression in tumor tissues and serum AFP levels, as found in the univariate and multivariate analysis. This histopathological finding is related to the aggressiveness of the tumor and a worse prognosis [25, 28, 29]. Brian Carr and Guerra (2016) [30] found a considerable difference in survival between patients with elevated and low serum AFP levels associated with portal vein thrombosis.

EpCAM expression, associated with lower survival rates, has been described in patients with breast cancer [31–34], ovarian cancer [34, 35], gallbladder cancer [36], and clear cell renal tumors [34, 37]. It has been reported that EpCAM expression in the HCC is associated with a shorter survival and a worse prognosis [5, 9, 22, 23, 27]. According to the classification of Yamashita et al. (2008) [9], EpCAM+AFP+ and EpCAM-AFP+ tumors were correlated with worse prognosis, whereas EpCAM-AFP- proved to produce better prognosis and, contrary to expectations, EpCAM+AFP- correlated with the best prognosis. Bae et al. (2012) [27] found no association between EpCAM expression and patients' overall survival. However, these authors showed that the EpCAM+ phenotype was significantly associated with survival in T1 stage HCC patients. Despite the initial stage, the patients had lower survival, in both, univariate and multivariate analysis. Similarly, in the present study, positive expression of EpCAM was not related to overall patient survival, but we did not observe the same results for T1 stages.

EpCAM has been targeted in clinical trials using monoclonal antibodies in different types of cancer [38–41], and it is believed that this molecule represents a new target for HCC gene therapy. Studies demonstrate that small interfering RNA (siRNA) can be successfully used for gene silencing in vivo [42, 43]. Bae et al. (2012) [27] demonstrated that the silencing of the EpCAM gene significantly decreased the proliferative and invasive capacity of HCC cells. Since anti-EpCAM and/or siRNA antibodies can be easily synthesized, studies show a rational basis for therapeutic approaches in HCC [26, 27]. A bispecific T cell engager (BiTE) antibody recognizing EpCAM has also been developed for cancer treatment [44]. Zhang et al. [44] demonstrated that anti-EpCAM BiTE 1H8/CD3 is capable of redirecting T cells to eradicate HCC cells as well as CSCs of HCC* in vitro* and* in vivo*. These authors proposed that anti-EpCAM BiTE 1H8/CD3 is a promising agent for treating HCC with limited Gal-1 expression.

Potential antitumor agents acting in the expression of EpCAM have been described [45]. Lidamycin (an enediyne anticancer antibiotic) was able to reduce tumor initiating cells of hepatocellular carcinoma reducing stem cell markers expression, such as EpCAM, by inhibiting Wnt/*β*-catenin pathway activation. In* in vitro* and* in vivo* experiments, it suppressed EpCAM expression, reduced the proportion of EpCAM+ tumor cell,s and inhibited tumor formation [45].

Pimozide (psychotropic dopamine receptor antagonist) also appears to have similar effect on EpCAM+ tumors, disrupting the Wnt/*β*-catenin signaling pathway and reducing EpCAM expression [46]. By downregulating Wnt/*β*-catenin signaling and EpCAM gene and protein expression, Pimozide reduced cancer cellular proliferation and viability and increased apoptosis induction in HCC cells [46].

The present study reinforced the information that a small percentage of HCC expresses EpCAM and that those patients with a positive expression would most likely benefit from a specific target therapy, corroborating with the premise that patients should be selected before such treatment is indicated. However, an anti-HCC effect of EpCAM-directed antibodies has not yet been reported in clinical studies.

The identification of a subclass of HCC EpCAM positive tumors, which exhibits cancerous stem cell characteristics, has an important clinical significance, since these cells may be another option of target therapy. It is believed that serum levels of AFP associated with immunohistochemistry can be used as a guide in the selection of patients for target therapy and can contribute to the identification of patients with different prognoses and to the TNM staging system.

This study does have one key limitation; that is, the fact that EpCAM and AFP were expressed in a low percentage of tumors may be the reason why we were unable to obtain any statistical significance for the clinicopathological characteristics included in this study.

In conclusion, there is a positive association between the immunohistochemical expression of the molecular biomarkers EpCAM and AFP, as well as with serum AFP levels. The HCC diameter ≤ 5 cm was associated with EpCAM expression, while angiolymphatic invasion was associated with AFP expression. A considerable number of cases of EpCAM positive HCC patients would benefit from a specific targeted therapy.

## Figures and Tables

**Table 1:** Demographic and clinicopathological characteristics in patients with hepatocellular carcinoma.

**Variable**	**Descriptive Statistic (%)**	**P value**
**Gender**		
Female	13 (31.71)	0.019*∗∗*
Male	28 (68.29)	

**Age**		
Mean (Standard Deviation)	59.2 (9.5)	N/A
Median (IIQ)	59 (54;66)	
Minimum; Maximum	25; 75	

**Comorbidities**		
No	22 (53.65)	0.357
Yes	19 (46.34)	

**Etiology**		
Idiopathic	7 (17.07)	0.241
Alcohol	5 (12.20)	0.058*∗*
Virus B	12 (29.26)	0.528
Virus C	17 (41.46)	0.015*∗∗*

**Serum AFP (ng / mL)**		
Mean (Standard Deviation)	1864.1 (9266.3)	N/A
Median (IIQ)	59 (54;66)	
Minimum; Maximum	1.9; 59900	

**Serum AFP (ng / mL)**		
Less than 100	26 (63.41)	0.001*∗∗∗*
100 to 400	7 (17.07)	0.027*∗∗*
Greater than 400	8 (15.51)	0.061

**Surgical procedure**		
Minor hepatectomy	28 (68.29)	0.019*∗∗*
Larger hepatectomy	13 (31.71)	

**Nodule**		
Single nodule	32 (78.0)	<0.001
Multiple	9 (22,0)	

**Diameter**		
≤ 5 cm	23 (56.1)	0.357
> 5 cm	18 (43.9)	

**TNM Staging**		
I	18 (43.9)	0.150
II	17 (41.5)	0.268
III	6 (14.6)	0.011*∗∗*

**Angiolymphatic invasion**		
No	24 (58.5)	0.584
Yes	17 (41.5)	

**Histological differentiation**		
Well	19 (46.3)	0.077*∗*
Moderate	18 (43.9)	0.150
Poor	4 (9.8)	0.001*∗∗∗*

**AFP in HCC**		
Negative	29 (70.7)	0.008*∗∗∗*
Positive	12 (29.3)	

**EpCAM in HCC**		
Negative	29 (70.7)	0.008*∗∗∗*
Positive	12 (29.3)	

Note: a homogeneity test was performed between the categories; *∗* p <0.10; *∗∗* p <0.05; *∗∗∗* p <0.01.

**Table 2:** Association between the immunohistochemical expressions of EpCAM and AFP in hepatocellular carcinoma.

**AFP**	**EpCAM**		**Total**	**P value**
	Negative	Positive		
Negative	25 (86.2%)	4 (13.8%)	29 (100.0%)	
Positive	4 (33.3%)	8 (66.7%)	12 (100.0%)	0.002*∗*
**Total**	29 (70.7%)	12 (29.2%)	41 (100.0%)	

*∗*: statistical significance measured by Fisher's exact test.

**Table 3:** Association between clinicopathological variables and expression of alpha-fetoprotein in hepatocellular carcinoma.

**Variables**	**Alfa-fetoprotein in HCC**		**Total**	**P value**	**EpCAM in HCC**		**Total**	**P value**
	Negative	Positive			Negative	Positive		
**Etiology**								
Idiopathic	6 (85.71%)	1 (14.29%)	7 (100.0%)		6 (85.71%)	1 (14.29%)	7 (100.0%)	
Ethanol	3 (60.00%)	2 (40.00%)	5 (100.0%)	0.703	4 (80.00%)	1 (20.00%)	5 (100.0%)	0.673
Virus B	8 (66.67%)	4 (33.33%)	12 (100.0%)		7 (58.33%)	5 (41.67%)	12 (100.0%)	
Virus C	12 (70.59%)	5 (29.11%)	17 (100.0%)		12 (70.59%)	5 (29.41%)	17 (100.0%)	

**Node**								
Single node	23 (71.88%)	9 (28.12%)	32 (100.0%)	0.999	22 (68.75%)	10 (31.25%)	32 (100.0%)	0.702
Multiple	6 (66.67%)	3 (33.33%)	9 (100.0%)		7 (77.78%)	2 (22.22%)	9 (100.0%)	

**Diameter**								
≤ 5 cm	14 (60.87%)	9 (39.13%)	23 (100.0%)	0.171	13 (56.52%)	10 (43.48%)	23 (100.0%)	0.038*∗*
> 5 cm	15 (83.33%)	3 (16.67%)	18 (100.0%)		16 (88.89%)	2 (11.11%)	18 (100.0%)	

**TNM Staging**								
I	15 (83.33%)	3 (16.67%)	18 (100.0%)		13 (72.22%)	5 (27.78%)	18 (100.0%)	
II	11 (64.71%)	6 (35.29%)	17 (100.0%)	0.241	11 (64.71%)	6 (35.29%)	17 (100.0%)	0.728
III	3 (50.00%)	3 (50.00%)	6 (100.0%)		5 (83.33%)	1 (29.27%)	6 (100.0%)	

**Angiolymphatic invasion**								
No	20 (83.33%)	4 (16.67%)	24 (100.0%)	0.045*∗*	18 (75.00%)	6 (25.00%)	24 (100.0%)	0.507
Yes	9 (52.94%)	8 (47.06%)	17 (100.0%)		11 (64.71%)	6 (35.29%)	17 (100.0%)	

**Degree of histological differentiation**								
Well	15 (78.95%)	4 (21.05%)	19 (100.0%)	0.492	16 (84.21%)	3 (15.79%)	19 (100.0%)	0.182
Moderate	11 (61.11%)	7 (38.89%)	18 (100.0%)		11 (61.11%)	7 (38.89%)	18 (100.0%)	
Poor	3 (75.00%)	1 (25.00%)	1 (100.0%)		2 (50.00%)	2 (50.00%)	4 (100.0%)	

**Serum alfa-fetoprotein (ng / mL)**								
Up to 100	23 (88.46%)	3 (11.53%)	26 (100.0%)	0.003*∗*	21 (80.76%)	5 (19.24%)	26 (100.0%)	0.006*∗*
100 a 400	3 (42.85%)	4 (57.14%)	7 (100.0%)		5 (71.42%)	2 (28.58%)	7 (100.0%)	
> 400	3 (37.5%)	5 (62.50%)	8 (100.0%)		3 (37.50%)	5 (62.50%)	8 (100.0%)	

*∗*: statistical significance by Fisher's exact test.

## Data Availability

The data for this research were obtained from the HCC patients assisted in the Hepatopancreatobiliary Service of the Alfa Institute of Gastroenterology, Clinical Hospital, Faculty of Medicine, Federal University of Minas Gerais, Brazil. The anonymized data can be found at the following link: https://www.dropbox.com/s/0ayplqbwpn8ygg4/HCCDATA.xlsx?dl=0, or from the corresponding author upon request.
